# Use of the theoretical domains framework and behaviour change wheel to develop a novel intervention to improve the quality of multidisciplinary cancer conference decision-making

**DOI:** 10.1186/s12913-020-05255-w

**Published:** 2020-06-24

**Authors:** Christine Fahim, Anita Acai, Meghan M. McConnell, Frances C. Wright, Ranil R. Sonnadara, Marko Simunovic

**Affiliations:** 1 Department of Health Research Methods, McMaster University, Evidence and Impact, Hamilton, ON Canada; 2 Johns Hopkins University, Bloomberg School of Public Health, Hampton House, Room 663, 624 N Broadway, Baltimore, MD 21205 USA; 3 Department of Surgery, McMaster University, Hamilton, 1280 Main Street West, Hamilton, ON L8S4L8 Canada; 4 Department of Innovation in Medical Education, University of Ottawa, 451 Smyth Rd, Ottawa, ON K1H 8M5 Canada; 5 Department of Surgery, Sunnybrook Health Sciences Centre, University of Toronto, Toronto Room T2 057, 2075 Bayview Ave., Toronto, ON M4N 3M5 Canada

**Keywords:** Theoretical domains framework, COM-B behaviour change wheel, Intervention design, Multidisciplinary tumor board, Multidisciplinary cancer conference, Multidisciplinary decision making, Cancer, Qualitative research, Knowledge translation

## Abstract

**Background:**

Multidisciplinary Cancer Conferences (MCCs) are prospective meetings involving cancer specialists to discuss treatment plans for patients with cancer. Despite reported gaps in MCC quality, there have been few efforts to improve its functioning. The purpose of this study was to use theoretically-rooted knowledge translation (KT) theories and frameworks to inform the development of a strategy to improve MCC decision-making quality.

**Methods:**

A multi-phased approach was used to design an intervention titled the KT-MCC Strategy. First, key informant interviews framed using the Theoretical Domains Framework (TDF) were conducted with MCC participants to identify barriers and facilitators to optimal MCC decision-making. Second, identified TDF domains were mapped to corresponding strategies using the COM-B Behavior Change Wheel to develop the KT-MCC Strategy. Finally, focus groups with MCC participants were held to confirm acceptability of the proposed KT-MCC Strategy.

**Results:**

Data saturation was reached at *n* = 21 interviews. Twenty-seven barrier themes and 13 facilitator themes were ascribed to 11 and 10 TDF domains, respectively. Differences in reported barriers by physician specialty were observed. The resulting KT-MCC Strategy included workshops, chair training, team training, standardized intake forms and a synoptic discussion checklist, and, audit and feedback. Focus groups (*n* = 3, participants 18) confirmed the acceptability of the identified interventions.

**Conclusion:**

Myriad factors were found to influence MCC decision making. We present a novel application of the TDF and COM-B to the context of MCCs. We comprehensively describe the barriers and facilitators that impact MCC decision making and propose strategies that may positively impact the quality of MCC decision making.

## Background

Multidisciplinary Cancer Conferences (MCCs) are regular meetings held by cancer specialists to prospectively discuss treatment plans for patients with cancer. MCCs typically include representation by surgeons, medical and radiation oncologists, pathologists and radiologists, and may also include nurses and other allied professionals. MCCs strengthen collaboration and communication among participating physicians, increase adherence to clinical guidelines, decrease wait times to treatment implementation, and improve multidisciplinary collaboration among cancer specialists [1–3]. Despite the importance of MCCs to the quality of care received by patients with cancer, numerous barriers to optimal MCC functioning have been described including gaps in case organization, teamworking, leadership and technology [3, 4]. Despite these identified gaps, there has been little research on the impact of interventions to improve the quality of MCC functioning. A concerted effort in one jurisdiction in the United Kingdom led to only slight improvements in MCC decision making quality [5, 6].

In 2010 in Ontario, Canada, Cancer Care Ontario (CCO), the agency responsible for promoting quality care for patients with cancer in the province, mandated the use of MCCs in hospitals. CCO set a target that required at least 80% of hospitals in the province to meet the required provincial MCC standards by 2015 [7]. A secondary goal was to double the volume of cases discussed by the 2015 deadline (from 24,000 cases in 2010 to 50,000 cases in 2015). The province met these goals, and by 2015, 80% of hospitals (51/64 hospitals) met the minimum CCO quality criteria. Recent data also show a further increase in MCC standard concordance among non-regional cancer center hospitals, with 85% of these hospitals having met CCO quality criteria [8].

However, there remain two challenges associated with MCC functioning: inadequate access to MCCs and lack of quality assessment of MCCs. Ontario data suggests that less than half of all new cancer cases are discussed prospectively in a multidisciplinary forum [8]. Resource barriers, specifically limited review time coupled with high case volumes, preclude MCC teams from discussing all new or suspected cancer cases. For instance, a study by our group demonstrated that only 20% of thoracic cancer cases at a leading academic institution were discussed at a MCC [9]. Second and perhaps more importantly, and similar to other jurisdictions in the world, there has been no formal retrospective or prospective evaluation of MCC decision making quality at the individual case or round/session level. Evaluations of MCC quality in Ontario have been limited to the types of specialists in attendance and MCC frequency. Such measures do not interrogate the quality of decision making or resulting treatment recommendations. Previous research by our group evaluating MCC decision making quality in a limited setting found gaps in the quality of information presented and the quality of MCC teamworking [10]. Via an evaluation of two MCCs, we identified variation in amount and quality of contribution by specialty, gaps in leadership, and little consideration of patient views during MCC discussion [10]. Informed by these data, we aimed to use Knowledge Translation (KT) methods to determine the barriers and facilitators to optimal MCC decision making and to identify potentially effective solutions to mitigating and leveraging these barriers and facilitators, respectively.

KT is the process of developing, disseminating and applying evidence to improve processes and outcomes. Examples of KT interventions (or implementation strategies) include the use of audit and feedback, incentives, educational meetings, and team training [11–15]. KT interventions are believed to be most effective if they first identify the determinants of behaviour change, and subsequently identify strategies that target these determinants [16]. To facilitate this, KT experts have increasingly called for the use of theoretical frameworks and models.

For this study, we were interested in developing a KT strategy that could potentially improve the quality of MCC decision making in an Ontario context. We report herein key results and observations. The intended audiences for this work include implementation scientists, researchers and clinician scientists interested in the pragmatic application of KT methodology and multidisciplinary cancer teams interested in the barriers, facilitators and potential solutions to improve MCC decision making.

## Methods

### Theoretical underpinnings

The Theoretical Domains Framework (TDF) and Capabilities-Opportunities-Motivation (COM-B) Behaviour Change Wheel were used to guide this study [17, 18]. The TDF can be used to identify factors impacting the uptake of a behaviour of interest, while the COM-B facilitates mapping of these factors corresponding interventions. There are only a few examples of studies that describe use of the TDF and COM-B to design KT interventions [19, 20].

The use of theory in intervention design is important, as it allows for the systematic mapping of behavioural constructs to corresponding KT intrventions. However, given the many theories of behaviour change, it is difficult to justify the prioritization of one theory over another. One benefit of the TDF is that it is a meta-framework that incorporates 33 psychology theories and over 128 behavioural change constructs. Additionally, the TDF can be directly transposed on to the COM-B Behaviour Change Wheel, thereby removing much of the ‘guesswork’ required to determine corresponding implementation strategies.

### Study design

We used a multi-staged approach to design the KT-MCC Strategy. First, key informant interviews based on the TDF were conducted with Ontario MCC participants to identify factors to optimal MCC decision making. We defined ‘optimal decision making’ as encompassing best practices for MCC case presentation, discussion and final selection of treatment plans. Factors were then linked to barrier and facilitator TDF-domains. Second, the COM-B model facilitated the mapping of identified TDF-domains to specific KT interventions, to create our prototype KT-MCC Strategy [18, 20]. Finally, focus groups with MCC participants were held to a) confirm the clinical relevance of the key informant data (i.e., trustworthiness); b) identify potential additional barriers and facilitators to optimal MCC decision making; and c) consider the acceptability and appropriateness of the proposed KT-MCC Strategy [21].

This study was approved by the Hamilton Integrated Ethics Review Board.

### Setting

The province of Ontario, Canada (population 13 million) is comprised of 14 geographic Local Health Integrated Networks (LHINs). Relevant stakeholders from two LHINs were sampled for this study. These two LHINs contain academic and community hospitals and collectively service a population of 2.5 million Ontarians, or approximately 20% of residents in the province.

### Part 1 – key informant interviews

#### Interview guide

The TDF includes 14 domains that may influence a particular behavior of interest. These domains include: *Knowledge; Skills; Social/Professional Role and Identity; Beliefs about Capabilities; Optimism; Beliefs about Consequences; Reinforcement; Intentions; Goals; Memory, Attention and Decision Processes; Environmental Context and Resources; Social Influences; Emotion;* and *Behavioural Regulation* [17].

We used relevant MCC literature to design content-specific questions relating to each of the 14 TDF domains. A content expert was consulted to ensure face validity of the interview guide (see Additional File 1). We used semi-structured interviewing which is recommended for interviewing participants in a single interview [22]. It allows the researcher to pursue probing questions to gain further context and ensure a comprehensive understanding of the key informants’ experiences or perceptions. The interview guide was modeled after examples provided in the implementation science literature [23–26].

#### Study procedure and participants

MCC standards outlined by CCO identify the following individuals as core members of the MCC: designated MCC chair and coordinator, and representatives from medical oncology, radiation oncology, surgery, pathology and radiology. Participation by nursing is encouraged, although not mandated, and was less prevalent at the study sites. MCC chairs, surgeons, medical and radiation oncologists, pathologists and radiologists were therefore prioritized as the target population for the key informant interviews. Participants were recruited using purposeful and snowball sampling. Participants were emailed and invited to participate in either a face-to-face or telephone key informant interview. An experienced qualitative researcher trained in the use of the TDF conducted all interviews. Following participant consent, all interviews were recorded and transcribed verbatim. We ensured participant representation by specialist type (surgeons, oncologists, radiologists & pathologists) to allow for a comparison of findings by specialist group.

Sample size was determined using Francis et al.’s framework for theoretically-rooted interviewing, which sets an a priori minimum sample size of 10, with a ‘stopping criterion’ when three interviews do not identify new themes (i.e., minimum of ≥13 interviews, given the last three interviews reach data saturation) [27].

#### Analysis

Deductive and inductive coding were used. First, a coding tree with context-specific definitions for each of the 14 TDF domains was developed and used to guide the deductive coding. Two reviewers then organized the interview data using the TDF domains. Next, open-coding was used to identify salient themes within each of the TDF domains. Themes are qualitative descriptions of interview findings that further provide context to the TDF domains. The decision to present the findings as both TDF domains and emergent themes was made to provide context to the theoretical findings and ensure that the key informant data were meaningful to the target population [28].

Two researchers first jointly analyzed three interviews to ensure consistency in coding. The two researchers double-coded the remainder of the key informant interviews. In instances where discrepancies in coding arose, the researchers discussed the differences in order to reach consensus. If a quote was found to fit into multiple themes or domains following discussion, it was double coded. Double coding allowed our research team to identify linked themes and related domains. The decision to double code the qualitative data is consistent with evidence that some TDF domains measure a combination of factors and do not represent an isolated behaviour [29, 30]. Salient quotes were highlighted to provide “thick descriptions” of participants’ experiences in MCCs. Finally, data were analyzed by physician specialist group.

### Part 2 – TDF to COM-B mapping process

The COM-B integrates 19 behavioural change frameworks into three facets of behavior change – capabilities, opportunities and motivation [18]. At the core of the COM-B are six sources of behaviour that affect the capabilities, opportunities or motivation needed for behaviour change including social and physical opportunity, automatic and reflective motivation, and psychological and physical capability [18]. These sources are linked to nine intervention functions (e.g., education, training) that can be used to potentially alter the behaviour. At the outer edge of the wheel are policy categories (e.g., environmental/social planning, legislation) that promote organizational change [18]. The intent of the COM-B is to facilitate the selection of KT interventions that are most likely to overcome identified barriers to behaviour change.

TDF domains identified as mediators to MCC decision making were mapped to the COM-B to first identify the facet of behaviour (capability, opportunity or motivation) requiring intervention. Then, corresponding intervention functions for each identified facet of behaviour were identified. Evidence regarding the efficacy of these intervention functions (gathered via a review of the Effective Practice and Organization of Care strategies, systematic reviews and evidence on teamworking from the business literature) was used to operationalize the intervention functions [13–15]. The interventions were further refined following discussions with focus group participants and presented as the first iteration of the KT-MCC Strategy [31].

### Part 3 – focus groups

#### Focus group participants

Three specialty-specific focus groups were held. The first focus group involved surgeons, the second involved medical and radiation oncologists, and the third involved radiologists and pathologists. Each focus group included a MCC coordinator. The MCC coordinator (who is sometimes a nurse or nurse practitioner) is responsible for organizing all MCC data and imaging prior to discussion, and to record the subsequent treatment recommendations. Focus groups took place at a single academic hospital site. Participants took part in either the key informant interviews or focus group discussions.

#### Study procedure

Focus groups with MCC participants were held to confirm the qualitative validity of the key informant data and to identify potential additional barriers and facilitators to optimal MCC decision making not identified in the key informant interviews. Qualitative validity pertains to the accuracy and relevance of findings, and is often referred to as trustworthiness or confirmability [32–35]. Focus group participants also considered the acceptability and appropriateness (as defined by Proctor et al.) of the prototype KT-MCC Strategy [21]. Insights obtained from the focus group findings were used as needed to modify the KT-MCC Strategy.

#### Data analysis

All focus group data were recorded and transcribed verbatim. Data were analyzed thematically using a compare and contrast method on Nvivo software. In this method, themes are compared in order to identify similarities and differences [36, 37].

#### Reflexivity statement

The primary author was trained in KT methodology and led the design of the discussion guides, under the guidance of MS and RRS. The primary author (CF) conducted the interviews and focus groups and holds extensive experience in these methods. CF and AA independently coded the qualitative data. Both researchers were trained in qualitative methodology and conducted the research as graduate students. The researchers did not hold a relationship with the participants prior to the key informant interviews or focus groups.

## Results

### Participant demographics – key informant interviews

Interviews were conducted from April 2016 – July 2017. Data saturation was reached at *n* = 21 interviews. Participants included seven surgeons, eight oncologists (five medical oncologists, three radiation oncologists), three radiologists and three pathologists. Participants were from three academic and four community sites. Participants reported attending MCCs for gastrointestinal (*n* = 14), hepatobiliary (*n* = 7), lung (n = 7), breast (*n* = 3), neuroendocrine (n = 2), genitourarinary (n = 2), sarcoma (n = 1), and gynecologic (n = 1) disease. Many participants attended multiple MCCs. The majority of interviews (17/21) were conducted in person, while the remainder (4/21) were conducted via telephone. Mean time per interview was 33 min (range 19 min – 78 min).

#### Barriers to optimal MCC decision making

Twenty-seven themes were identified as barriers and ascribed to 11/14 TDF domains. Most domains contained 2–4 qualitative themes (see Table 1).

**Table 1:** Barriers to Optimal MCC Decision Making

TDF DOMAIN	THEMES	QUOTES
**Knowledge**	Lack of awareness of CCO (governing body) guidelines regarding which cases should be discussed at MCC	“I didn’t even know CCO guidelines existed” – P16
	The quality of the discussion and decision making process is contingent on the amount of knowledge the MCC participants hold	“I’ve spend forty-five minutes at looking at the patient’s imaging having partial information … [this discussion is] kind of poor quality or sub-par, it has no place there. The level should be higher than that. – P3
**Memory, Attention, Decision Processes**	MCC chairs do not always control the flow of MCC discussion	“It gets frustrating because the discussion goes on way past the decision making point and we going to get into “at nauseum” discussions where there cannot be a black and white answer ... the discussion is out of line” – P8
	Presenting physicians (MRP) are not prepared for MCC discussion	“I mean, we’re all players in there, they [surgeons] ask us [diagnostic imaging] to prepare for those rounds. I think it’s unacceptable that they’re not prepared for the rounds, you see what I mean? If they have two patients that they, at least for their patient, they have to know everything, and they should review [the reports]” – P3
	There is no standard format for presentation of cases or processes of discussion/decision making	“What had been happening is we would print out the case list, me or, generally the nurse, would scribble the decision, and it would get put in a binder, and once again they get thrown out. So there was no way to even go back and document “Oh, this person’s been discussed on three different occasions,” or to pull up the last discussion” –P3
	The right specialists are not in the room at time of MCC discussion (linked to time demands) - Practice site (community, academic) influences attendance and subsequently, decision making	“We’re limited in doing the multidisciplinary rounds by, usually by surgeons’ availability, because their time is [limited], we can’t really meet at lunch because they’re in the O.R. and, so, you know, we’re limited by that” –P4 “I’m in a difficult spot because I’m the sole oncologist [at a community site], so I have to attend so many MCC, but most people at academic centres, you’re one of 15 oncologists, so if you don’t show up, one of your colleagues will” – P1
	Decisions vary by the individuals present - Hierarchy (age, seniority) influences decision making	“[There is] definite variability based on who is in the room. So the most is in surgery ‘cause you know, one surgeon operates, and the other feels like they can’t operate, so there’s a lot of surgical variation” –P7 “or even though we try to do evidence-based, sometimes the trial that we base our evidence on is not, not the best, right, and some people will say ‘Well, I’ll still use that data’, and some people will say ‘Oh, I’ll throw it away’” – P1 “There’s standard of care, then there’s a bit of art to oncology, and there’s different ways that people do things” –P1
**Environment**	Group mandates to mitigate time demands (e.g., max number of cases, deadline to submit cases) are not always effective	“Part of the frustration with MCC is the turnaround time...So for example … you have to have your case emailed in by Wednesday, or whatever it is to get on for the next week...well I see my new patients on Wednesdays, right, and so I will often... say “Please, please [discuss my case]” – P2
	Inadequate administrative supports (community and academic sites)	“It’s a lot of legwork, that maybe academic [sites that have coordinators] don’t really appreciate, you know, but certainly I’m [med onc] the one who has to get that [imaging] disc, get it to the right person, make sure it’s uploaded, and sometimes I’ll go [to MCC] and my disc is not uploaded, so I can’t present [my case]” –P1 “I have no secretary, essentially. We have one on paper, and I ask her [to do] something and she [cannot] … seriously, no, no, I’m not joking, so we have no clerical help” –P3
	Inadequate physical resources (space, technology, access to imaging)	“The teleconferencing itself, it’s a complex process, sometimes it’s time consuming, we don’t always hear each other that well” –P6 “We have barriers here with our technologies so it takes forever to load up images...and the computers we use are too slow I think, they always seize up”-P9 “So you have a problem, you’re in the room, and then you have somebody knocking at the door and saying ‘We have the room at five o’clock, so please finish your rounds” –P3
**Social Influences**	Lack of soft skills (e.g., effective communication, collaboration) among group	“Groups, really, where people are not really collaborating...I mean, some people have pretty, can have pretty bad attitudes, and that’s known, right, and we have some rounds that work not as well as others for that reason” –P3 “We’re [a] pretty collegial group, so in our own environment there’s not much of a conflict, we can call each other idiots or swear but it’s very benign” –P12 “I was sitting around the table and [was able to] stop the side discussions and all the joking and all the irrelevant stuff but it’s much more difficult when you’re sitting here and you see them on TV [satellite site] making jokes and stuff” –P6
	Negative group dynamics/ Bullying	“I can certainly see in certain centres there may be bullying from one group to the other or from one physician to the other. It’s just like high school” –P12
	Lack of psychological safety (ie: ability to ask questions/ make mistakes)	“But the folks [at certain MCC] will be a major pain … they make you feel stupid … and I can name names of oncologists … who won’t go back to those rounds because they’re made to feel stupid at the rounds” –P2
	Certain individuals dominate the conversation	“Well everything is always driven by a few people but there is always an opportunity – no body is shut down. If they don’t speak, its because they choose not to” –P12 “[at some rounds] we have forceful individual who want to take over, want to shine” – P3
**Social/Professional Role and Identity**	The desire to discuss cases collaboratively at MCC is not tied to professional role/identity (i.e.: some physicians don’t feel that they must attend MCC in order to effectively fulfill their professional role)	“The impact of that [MCC] would be extremely minimal. If you’ve got a well-trained clinician, they can decide which [cases] need to be discussed” –P9
	Preference of ‘solo practice’ versus ‘collaborative style’ defines willingness to regularly attend MCC	“The other barrier to that is that surgeons are very proud and autonomous in the way that they want to perform in operation and they don’t want to take criticism very easily and so volunteering to subject yourself to scrutiny and criticism may not be very acceptable to a lot of surgeons” –P7 “I mean, the problem that I have with [not attending] is that I find it hard to believe that anyone in a large-volume centre that treats very complicated cases doesn’t have any cases where they need peoples’ help ... how could you be treating two-hundred people a year and not have questions on, like, 10% of them, I mean, it just doesn’t make any sense” – P14
	Professional identity (linked to specialty/ hospital site) and beliefs dictate treatment recommendations and preferences	“When [academic physicians] go out [to a community site], they [community physicians] get their backs up and they resent the fact that you’re the “professor” coming … and it’s like ‘Huff, you think you know more than me!’ – P14
**Emotion**	Emotions during MCC discussions can run high and lead to conflict	“There is definitely conflict” – P1 “Every once in a while, some good-old fights break out” – P2
	Feeling underappreciated; undervalued in the MCC decision making process	“Nobody, honestly, has the appreciation of the amount of time … we [radiologists] put in those rounds and the time it takes … no clue or no appreciation, or no idea, actually, how detailed and how, the amount of time we have to spend at looking, at looking at [the images] –P3 “The fact that they [radiologists] do as many MCC as they do with no direct compensation whereas everybody else in that room is [compensated] in some form. It’s not fair” – P8
	Emotion and recent experiences affect decision making	“The one thing that’s hard to capture is the mood that the physicians are in – there can be fluctuations in the mood where you can present the same case weekly three times, and get a different opinion depending on the mood of the specialists that may be involved in the decision making”–P12
**Beliefs about capabilities**	Capacity to make a decision is limited when there are conflicting decision recommendations	“You’ll see them arguing over, you know, long-course radiation chemo-radiation versus short-course, and sometimes whether you need any radiation pre-op if you do a good TME (total mesorectal excision). So as a non-surgeon, non-rad onc, it’s quite confusing when I make the referral and then we have two completely different opinions, and I think a lot of medical oncologists feel that same way, that on that particular issue, that we’re a bit lost” –P1
	Individuals use MCC to empower their own decisions	“In as much as that if something goes wrong, at least I can say ‘Well, it wasn’t just my decision, it was everyone’s’” –P16 “You know, if somebody attends tumor boards regularly and you like the way they think and their opinion then you’re more likely to want to work with them … And by virtue or referring to that person, you refer less to the persons that you don’t like” –P7
**Beliefs About Consequences**	There are little to no perceived negative consequences to individuals not participating in MCC discussion (as long as the hospital site meets the minimum provincial requirements)	“There’s unfortunately no consequence to not attending tumor board” –P12
	Perceived consequences around the impact of MCCs on patient care is positive	“I think there are advantages personally and I think there are advantages for the patient” –P4
**Behavioural Regulation**	Regulation takes place at a hospital level, and not individual level (theme correlates with beliefs about consequences)	“The surgeon wanted to do a radical prostatectomy, everyone, even all of the surgeons were like ‘No!’ like, “This is wrong’, and he did it anyways” –P14
**Reinforcements**	Carrots vs. Sticks: Beliefs that lack of ‘sticks’ is a barrier to efficient MCC discussion and decision making	“If people can’t comply [with MCC goals] you either say ok we’ll let inefficiency reign … and offenders will stay offenders … or you’re gonna say no were serious about this, therefore the rules are absolute” – P8 “I don’t believe that carrots help. Going to MCC and learning, it should be a carrot enough. So, I think, I think there’d have to be a stick. It would have to be, if you don’t show up, then you lose... ‘you lose money, you lose ability to see patients’, or whatever it is. You theoretically could give people a financial bonus to go but I have a philosophical problem with paying people for things they should be doing already” –P14 “It’s hard to police that though unless you have, you really would need a physician champion, who’s senior enough and has the authority to say “Well, that’s your question [which wasn't submitted according to protocol], we’re not reviewing that this week.” –P17
**Optimism**	Evidence of disfavor for CCO guidelines regarding which cases to discuss at rounds/ how MCC are evaluated	“Well their [CCO’s] intent is to try and encourage MCC to happen, I think they’re a little bit too prescriptive and they’re not practical for some disease sites and some institutions. Same thing with the sub-specialties that are required to be there, it is not always logical to have all the sub-specialties there. For instance, from the perspective of hepatobiliary rounds, radiation oncology is not usually all that common” –P9

Participants most commonly described barriers relating to MCC processes. For instance, participants identified gaps in MCC leadership, lack of core participant attendance, lack of MCC participant preparation, unstructured processes for case presentation, individual treatment preferences by treating physicians, and prolonged case discussions (see Table 1 for sample quotes). These themes interacted to impede MCC decision making. One participant stated,

*“[MCCs] get frustrating because the discussion goes on way past the decision making point and we get into ‘at nauseum’ discussions where there cannot be a black and white answer”- Participant 8 (Surgeon)*

Participants also commonly described themes relating to social dynamics that negatively impact MCC decision making including a lack of soft skills (e.g., use of non-effective communication strategies) among group members, negative group dynamics or bullying, the inability to ask questions openly, and the domination of the conversation by few individuals.

Other identified themes included a lack of: awareness of guidelines outlined by CCO regarding MCC processes, clinical knowledge required to meaningfully inform cases, and buy in regarding MCCs by certain physicians/specialty groups. Some participants also reported hierarchical group dynamics between academic and community sites, which impacted decision making. Contextual factors including limited time, administrative supports and physical resources (e.g., access to imaging) were also found to impede decision making. Such themes were often tied closely to barriers categorized in the *Emotion* domain, which included feelings of under-appreciation and conflict during the decision making process. For instance, feelings of under-appreciation arose when a MCC participant felt they had dedicated significant time to prepare for an MCC case, and their colleagues had not reciprocated in kind. This is best explained in the following quote by a radiologist who highlights the interaction between time demands and feelings of under-appreciation,

“*Nobody, honestly, has the appreciation of the amount of time … we [radiologists] put in those rounds and the time it takes … they [surgeons] ask us to prepare for those rounds. I think it’s unacceptable that they’re not prepared.” – Participant 3 (Radiologist).*

Lack of attendance by presenting physicians (oncologists or surgeons), and time barriers often led to a cancellation of cases listed for discussion. This further caused feelings of frustration for radiologists and pathologists (who had prepared the case imaging and slides), and other participants who were in attendance at time of MCC discussion. Additional themes are outlined in Table 1.

#### Identified barriers by specialist group

While some barriers were common to all specialists (e.g., time demands), others varied by specialist group. For instance, surgeons identified scheduling conflicts and operating room demands as barriers to regular MCC attendance. Surgeons’ level of participation in MCCs varied according to their intrinsic beliefs regarding the importance of collaborative decision making (i.e., those who valued collaborative decision making were more likely to regularly participate in MCCs). Moreover, surgeons reported that surgeon hierarchy, technical ability, and group dominance often influenced the final surgical recommendation.

Unlike surgeons, there was less variation in oncologists’ opinions regarding the role of MCCs, and most oncologists cited MCCs as an integral part of their practice. The most common barrier cited by oncologists was the inability to make a decision due to limited evidence and conflicting treatment recommendations. For instance, one medical oncologist highlighted being “quite confused” when surgeons and radiation oncologists presented conflicting treatment decisions. Oncologists further highlighted lack of leadership led to cyclical discussions without a clearly articulated final plan, as seen in the following quote,

*“The facilitator tends to just let people talk in circles over and over again for ten minutes about the same thing and there’s no directing the discussion to ‘Okay, let’s kind of get back on course to this patient … let’s kind of, you know, circle back to what do we need to do for this patient now.’”- Participant 16 (Medical Oncologist).*

Pathologists and radiologists described the significant amount of time required to prepare for MCCs as a barrier and highlighted that MCC preparation adds greatly to their regular workload. For instance, preparation of radiologic or pathologic findings for a MCC can take up to one full work-day to prepare. These time barriers led to subsequent feelings of frustration and under-appreciation when other treating physicians did not adequately contribute the required time to prepare for, and attend, MCCs.

#### Facilitators to optimal MCC decision making

Thirteen themes ascribed to 10/14 TDF domains were identified as facilitators to optimized MCC decision making. As expected, the converse of many barrier themes were identified as facilitators. For example, physician knowledge of available clinical guidelines and recent evidence facilitated clinicians’ ability to make a treatment decisions; however, the absence of this knowledge was a barrier to decision making.

A summary of all identified domains and corresponding themes are listed in Table 2. The most commonly reported facilitators to optimized MCC decision-making included opportunities for learning and standardization of decision making and facilitated collegiality and teamworking. Mandated MCCs and buy-in from leadership were also facilitators in this context.

**Table 2:** Facilitators to Optimal MCC Decision Making

TDF DOMAIN	THEMES	QUOTES
**Knowledge**	MCC provide opportunities for learning (from colleagues, other specialists, resident learning)	“We learn way more in MCC than anything else now” – P14 “You learn things from the MCC, right, a trial might come up that you weren’t aware of, or a new drug approval might come that you didn’t know about, and you get that education at the tumour boards” – P1
	MCC allow for standardization of decision making and treatment plans	“You get to stay up with what the rest of your colleagues are thinking, and we get some kind of standardization around treatment” –P4
**Skills**	Attending MCC allows specialists to better collaborate with their colleagues/ understand what others need	“From my perspective as a pathologist, I have learned a lot over the years about what is relevant and what is not … what are the major parameters that radiation oncologists, medical oncologists or the surgeons look for in guiding their management” – P15 “I love getting to see how my other colleagues think, we don’t get an opportunity to do that outside of MCC, because right, it’s not like I go to my colleagues’ clinics, so right, it’s good to know how other people are thinking” –P1
**Social Influences**	The ability to work cohesively as a group positively impacts decision making and teamworking	“You have to be able to practice as a group and to value people’s opinion” – P3 “Culture eats strategy for breakfast … in a big group inter-personal relationships are diluted but in a smaller group, inter-personal relationships are very important. And so all of that will probably overshadow any process [of decision making]” – P7
	MCC facilitate collegiality	“There’s coffee and muffins there...and there’s, every one of the surgeons, they like to come and talk about their cases, like I think it’s a very positive social culture” – P10
**Social/Professional Role and Identity**	Many MCC participants feel a personal responsibility to discuss cases, beyond the scope of CCO instruction (e.g., use of email to circumvent time restraints)	“I get emails all the time. They’ll email like ten experts...and we all weigh in on how we might look at a case” –P2 “Email is also quite good because for a lot of non-urgent things you can just send an email and get responded to later down the line” – P11
**Beliefs about capabilities**	Participation in MCC doesn’t limit physician autonomy to make decisions	“I don’t think [autonomy is affected], because at the end of the day, we’re making recommendations, and it’s not like they have to follow through with them if they are uncomfortable or if they don’t agree” – P2 “I don’t feel that my autonomy has been taken away from me, because I probably would have been thinking about the problem differently” –P7
**Goals**	MCC groups have set goals to improve efficiency and ensure comprehensive discussion of cases - Rotate attending specialists - Set limits on number of cases to be discussed - Triage cases based on urgency MCC goals dictate what MCC participants define as an ‘optimal MCC’	“We rotate, so we send a medical oncologist to [rounds] every week” – P2 “We try to time the discussion as well with patient’s treatment urgency, kind of, sense of urgency, we triage the cases” – P3
**Intentions**	Motivation to discuss cases due to: - Patient requests - Intrinsic motivation - Facilitating quality improvement	“There are people who bring cases to tumor board when they tell the patient up front we will discuss you at tumor board and then we will come up with a recommendation” – P12 “For the support and care of my patients. I’ve always gone [to MCC], I’ve been doing these for almost 20 years” – P9
**Optimism**	General positive attitudes towards MCC	“They’re [MCC] absolutely vital. I can’t imagine having a centre where you were having any area where there’s multidisciplinary care of patients where you’re not getting together to discuss the difficult cases” – P14
**Beliefs About Consequences**	Positive consequences of MCC decisions: - streamlines decision making for complex cases - saves patients from unnecessary consults/ results in more efficient care - improves quality care	[If someone says] “well, actually they should probably see this specialist,” and then they [patient] wait for a consult to see that specialist, I mean that’s avoided, the waiting from one doctor to another, so I think that’s its primary benefit, is that you can narrow down what to do fairly quickly and were the patient should go next “– P16
**Reinforcement**	CCO ability to withdraw funding is a major driver in bringing patients forward for MCC discussion	“There’s a [CCO] score card, and one of them has to do with, um, participation in MCC, so, you know … you had to have five [MCCs] per quarter … there’s also pressure from the organization to make sure that the organization gets credit, because if they don’t, then there are funding implications” – P4 “CCO will say, ‘you did twenty radical prostatectomies last quarter, only four of those patients saw a radiation oncologist, can you explain why?’ And with the subtle hint that eventually, they’ll start to withdraw funding if the patient’s aren’t seen” –P14 “We have to meet this metric otherwise Cancer Care Ontario will take our money away” – P16
	Personal Incentives: - Billing for MCC - Obtaining continuing medical education credits	“I think people like going to rounds, I think it’s an enjoyable experience generally, I can’t see the financial being the driving force but I mean well you have that for sure” –P11

The TDF domains of *Skills, Intentions,* and *Goals* were identified uniquely as facilitators to MCC processes. Participants reported that regular attendance and participation in MCC rounds increased their knowledge and skills, and allowed them to gain an appreciation of their colleagues’ decision making practices. For example, pathologists highlighted that participation in MCCs equipped them to better present histological findings to treating physicians, while simultaneously promoting a collaborative culture of interdisciplinary teamwork. Motivations to engage in MCCs stemmed from an intrinsic affinity to quality improvement. Intrinsic motivation and general optimism towards MCCs were suggested to be more effective than external reinforcements/penalties.

Additional motivators to optimize MCC decision making quality included patient preference to have their case discussed by a multidisciplinary team, a desire to collaborate with peers, and increased confidence in the final treatment decisions. Many participants described the importance of streamlined MCC processes (e.g., attendance schedule and a triage system for urgent cases) to facilitate decision making. Recommendations on operationalization of these processes differed based on the culture and needs of the individual team.

#### Identified Facilitators by Specialist Group

We did not identify any major significant differences in identified MCC facilitators by specialist groups. Rather, MCC participants generally spoke favorably of MCCs, and noted their importance to patient care, as demonstrated in the following quote,

*“[MCCs] are absolutely vital. I can’t imagine having a centre [or] any area where there’s multidisciplinary care of patients, where you’re not getting together to discuss the difficult cases” – Participant 14 (Radiation Oncologist).*

Some radiologists suggested that they did not receive the same level of monetary compensation as other MCC participants. However, reimbursement inequalities did not appear preclude radiologists and pathologists from regularly attending and participating in MCCs, which further reinforces the notion that intrinsic motivation is likely the main catalyst to MCC participation.

### Corresponding interventions to barriers and facilitators

The COM-B mapping exercise revealed that all six sources of behaviour change on the behaviour change wheel (psychological and physical Capabilities; social and physical Opportunities; automatic and reflective Motivation) could benefit from potential intervention. Based on these data, we determined that a multipronged intervention was required to improve the quality of MCC decision making. Table 3 outlines TDF domains identified as barriers or facilitators to MCC decision making and depicts the corresponding COM-B behavioural facet and intervention functions. Identified COM-B intervention functions to improve MCC decision making included modeling, environmental restructuring, restrictions, education, persuasion, incentivisation, coercion, enablement, and training. Table 4 presents the evidence and rationale that guided the formation of pragmatic implementation strategies.

**Table 3:** TDF Domains Mapped to COM-B

**COM-B SYSTEM**	**TDF DOMAIN**	**INTERVENTION FUNCTION**^**b**^
Capability: Psychological	1. Knowledge 2. Memory, Attention and Decision Process 3. Behavioral Regulation	**Education:** Increasing knowledge or understanding **Training:** Imparting skills **Enablement:** Increasing means/reducing barriers to increase capability or opportunity
Capability: Physical	4. Skills^a^	**Training** **Enablement**
Opportunity: Social	5. Social Influences	**Restriction:** Using rules to increase the target behaviour by reducing the opportunity to engage in competing behaviours **Environmental Restructuring:** Changing the physical or social context **Enablement:** Increasing means/reducing barriers to increase capability or opportunity
Opportunity: Physical	6. Environmental context and resources	**Restriction** **Environmental restructuring** **Enablement**
Motivation: Automatic	7. Emotion	**Persuasion:** Using communication to induce positive or negative feelings or stimulate action **Incentivisation:** Creating expectation of reward **Coercion:** Creating expectation of punishment or cost **Modeling:** Providing an example for people to aspire to or imitate **Enablement:** Increasing means/reducing barriers to increase capability or opportunity **Environmental Restructuring**
Motivation: Reflective	8. Professional role and identity 9. Beliefs about capabilities 10. Goals^a^ 11. Intentions^a^ 12. Beliefs about consequences	**Education** **Persuasion** **Incentivisation** **Coercion**
Other (not transposed on TDF)	13. Reinforcement 14. Optimism	

^a^TDF domain uniquely identified as a facilitator to MCC discussion and decision-making

^b^Definitions taken from Michie M, van Stralen M, West R. The behaviour change wheel: A new method for characterising and designing behaviour change interventions. *Implementation Science*, 2011; 6:42

**Table 4:** KT-MCC Strategy – Intervention Components

KT-MCC Strategy Intervention	Intervention Details	TDF Domains	COM-B Intervention Functions	Rationale/ Evidence
**Workshops to develop local consensus processes**	- Participants will be presented with data regarding the functioning of their own MCC in a didactic session - Participants will then be guided to select local consensus processes regarding team purpose and goals - Consensus process will include expectations for: weekly attendance, case submission process, processes of discussion, and MCC documentation, and ‘carrot vs stick’ approach to reinforcement of processes	- Memory, Attention and Decision Processes - Behavioural regulation - Social influences - Environmental context and resources - Goals - Knowledge - Skills	- Environmental restructuring - Education - Persuasion - Restrictions - Coercion	PARTICIPANT FEEDBACK - MCC processes differ by MCC team - Goals for MCC team differ depending on the context of the team and the nature of the disease site EVIDENCE - Workshops are optimized when they involve interactive and didactic sessions [14, 38, 39] - Tailored messaging improves adherence to behavioural interventions [40]
**Team Training**	- Team training session led by a team training expert. - Expert will provide MCC participants with actionable recommendations to improve MCC teamworking and soft skills	- Social Influences - Emotion - Skills - Memory, Attention, and Decision Processes	- Training - Environmental restructuring - Enablement - Education - Persuasion	PARTICIPANT FEEDBACK - Gaps in MCC decision making that stem from a lack of ‘soft skills’ EVIDENCE - Training to promote teamworking (i.e., soft skills) as opposed to taskwork (e.g., technical skills) more significantly impacts process outcomes - Business teamworking literature highlights a balance between ‘speaking up’ and ‘listening intensely” [41] - Participants should feel comfortable to ask questions, make mistakes (psychological safety) without negative repercussions[42] - Members should speak freely and challenge status quo [41–45]
**MCC Chair Training**	- MCC chairs act as gatekeepers to the success of the KT-MCC- MCC chairs as opinion leaders may influence MCC participant behaviour - Chairs will be invited to participate in a training session with a team training expert who will outline strategies to promote effective discussion, teamwork and efficiency during decision making - The research team will partner with MCC chairs to allow for further KT-MCC Strategy tailoring/ intervention selection	- Memory, Attention and Decision Processes - Behavioural Regulation - Social Influences -Social/Professional Role and Identity	- Modelling - Environmental restructuring - Persuasion - Education - Training	PARTICIPANT FEEDBACK - Lack of MCC leadership found to negatively impact decision making - Gaps in leadership correlated with cyclical case discussions, unequal contributions by MCC participants, and unclear final treatment plans EVIDENCE - Use of opinion leaders in tandem with other interventions can successfully influence behaviour change [13]
**Standardized Intake Form and Synoptic Checklist**	- Ensuring preparedness at time of MCC discussion will likely promote discussion clarity and efficiency of decision-making - Submitting physicians will complete a standard intake form prior to the MCC round (e.g., define a clear clinical question, provide a summary of patient history, specify the relevant imaging/pathology required for case discussion) - Chairs will be given a synoptic reporting form to guide case discussion. The form will prompt the chair to ensure relevant information is considered and a final treatment decision is articulated	- Knowledge - Environment - Memory, Attention and Decision Processes - Beliefs about Capabilities - Beliefs about Consequences	- Environmental restructuring - Modelling - Training - Coercion	PARTICIPANT FEEDBACK - Lack of imaging at time of discussion, gaps in patient case history presentation and lack of preparation by presenting physician are barriers - Participants found MCC discussions confusing and organized, and were unsure of how to proceed with treatment - No perceived consequences to lack of participation EVIDENCE - The MDT-QuIC checklist [46] was found to be a useful tool for MCC case preparation, case discussion, and MCC decision records. - The synoptic reporting form will serve as a reminder prompt to chairs and teams [46]
**Audit and Feedback**	- Feedback on MCC decision making will be evaluated and fed back to participants - Chairs and teams will select the quality markers to be fed back by research team - Chair will disseminate feedback	- Knowledge	- Modelling	PARTICIPANT FEEDBACK - No feedback on MCC quality (apart from CCO metrics) provided to participants - MCC participants have little knowledge of current MCC quality EVIDENCE - Feedback is most effective when disseminated by a leader, provided on an iterative basis, provided both verbally and in writing, and includes clear targets and recommendations for improvement [47]

The resulting KT-MCC Strategy interventions derived from the mapping exercise included workshops, chair training, team training, standardized intake forms and synoptic checklist, and, audit and feedback [17, 18]. These strategies are further described in Table 4.

### Participant demographics – focus group

A total of *n* = 18 MCC participants took part in the focus groups (*n* = 5 surgeons; *n* = 6 oncologists; *n* = 7 radiologists and pathologists). Participants reported attending MCCs for gastrointestinal, hepatobiliary, genitourinary, lung, breast, and sarcoma disease.

Table 5 compares all key informant and focus group data. All key informant themes were confirmed by at least some focus group participants, and the focus groups did not identify any new barrier or facilitator themes. Consistent with the key informant data, focus group participants generally held positive attitudes regarding the benefits of MCCs (e.g., improved collegiality, timely development of treatment plans, and reassurance for the management of complex cases). They did not perceive incentives, such as billing or continued medical education (CME) credits, to be significant motivators to their MCC engagement. Barriers identified through the key informant interviews were confirmed.

**Table 5:** Key Informant and Focus Group Data

**TDF Domain**	**Key Informant Themes identified as Barriers**	**✖ group thinks this is not a barrier** ✔** group agrees this is a barrier** ☐ **group divided**			**Sample Quote**
		Surgery	Oncology	Diagnostic Specialists	
Memory, Attention and Decision Processes	Presenting physician not prepared	✖	☐	✔	“MCCs are complex and there are occasions where people present and they come to a conclusion and (then they) suddenly realize, ‘oh I missed this detail’”
	Chair does not control flow of discussion	✖	✔	✔	“Sometimes the chair forgets they’re the chair and gets involved in the discussion as one of the experts” “Sometimes we don’t get through all the cases because some people go on and on and the chair doesn’t control it”
	No standard format for case submission	✔	✔	✔	“very rarely does (the MCC form) get filled out adequately, some people don’t fill it out at all and their cases still get on MCC, then they don’t show up and we have to actually try to figure out what their question is” "at rounds they’re like ‘well what about this margin’ and you’re like ‘oh I can’t believe I have to go through the slides again'. It would be much easier if they told us ahead of time what the specific question was because you have to do a total review of the case, which could take hours”
	No standard format for case presentation	✔	✖	✖	“So I find that having a structure to the presentation so you know what is your question, a very brief history … this is the scan and this is the imaging … when people ramble you don’t even know what they’re asking you just lose focus and you probably don’t even get as much information.”
	The right specialists are not in the room	☐	✔	✔	“I’ve seen that … if nobody from rad onc happened to be there, then that case has to be skipped over and it can’t be discussed...that just completely limits the discussion”
	No documentation standards	☐	✖	n/a	“(At rounds) there is somebody writing and they will ask me, what did you say … .and this piece of paper, I’m like where does this paper go? I wish … sometimes I know some patients have been discussed and I don’t know where to get that information”
Environment	Inadequate time within MCC to discuss all desired cases	✖	✔	✔	“there is never enough time for anything, everything is pushed to the limits”
	Lack of time to prepare for MCC	✔	✔	✔	“There should be a little column to what the pathologic issue is (ie: what the specific question is), especially for pathology – radiology can scroll up and down in the films where have to look at slides. We can’t do it on the spot”
	MCC times limited by specialist availability	✔	✔	✔	“There is never a Monday at 12 o’clock (time of MCC) that I am free, so I would have to plan for well in advance and completely rearrange a weekly schedule to be available … it’s a tough (meeting) time that doesn’t consider the challenges surgeons face. If they put it Monday at 7 am or 5 am I would be there every week”
	Inadequate administrative supports	✔	✖	✔	“We have zero administrative support. Resources are an issue, that is a huge thing”
	Inadequate physical resources	✔	✔	✔	“Technology is always an issue … ..always … .a lot of wasted time – we definitely have inadequate support for that” “Someone broke the microscope, so we have no microscope. I don’t know whose responsibility it is to replace it but it hasn’t been there for eight months”
Social Influence	Ineffective communication	✔	✖	✔	
	Negative group dynamics	✔	✔	☐	“It’s petty … you know the personalities and what happens, so it can be uncomfortable”
	Bullying	☐	✖	✔	“I have been belittled at those rounds” “It’s to a point in this disease site where one of the surgeons walked out because of the way they were treated”
	Lack of psychological safety	✔	✔	✔	“We are supposed to come in like it’s a safe place and residents always feel like they are being judged”
	Inneffective communication/ Certain individuals dominate the conversation	✔	✔	✔	“whoever would yell the loudest was ultimately (listened to)”
Knowledge	Limited evidence to guide treatment plans	✖	✖	✖	“(A lack of evidence) enhances the discussion. Part of the impetus behind MCC in the first place is there is not a perfect answer for every single scenario”
	Conflicting recommendations (art of practice)	✖	✔	✔	“It’s the nebulous stuff … that makes it hard”
	Belief that use of email is equivalent to discussing case in MCC	✔	✖	n/a	“Email is not a sufficient way to discuss patients”
Goals	Lack of consensus regarding MCC purpose	✔	✔	✔	“This gets to the heart of why certain rounds may have more tension … it depends on whether you are obliged to have consensus (regarding treatment plan) at the end … if you are obliged to have a consensus things might be more heated because then you are bound to following through (on the plan) that you may not agree to”
Social/Professional Role and Identity	Intrinsic belief that participation in MCC is outside of the scope of treating physicians’ professional role	✖	☐	✖	“That option is not available as a (diagnostic specialist). You must be present” “Not all of the surgeons always attended”
Beliefs about Consequences	No consequences to physicians who do not attend or participate in MCC	✔	✔	n/a	“it’s a barrier when they put on a case and then they don’t show up”
**TDF Domain**	**Key Informant Themes identified as Facilitators**	**✖ group thinks this is not a facilitator** ✔** group agrees this is a facilitator** ☐ **group divided**			**Sample Quote**
		Surgery	Oncology	Diagnostic Specialists	
Social Influence	Strong collegiality and teamworking	✔	✔	✔	“those (rounds) are valuable to me and I think I speak for my colleagues … you are there to be collegial to your other colleagues … you are there because it is part of your role as a professional collaborator”
Environment	Standard case submission (intake form) increases efficiency	✔	✔	✔	“I think this is where you would have a stick, right. This is where you have to stick to say ‘if you do not meet this minimum standard for submission then the case isn’t going to get discussed … if you are not uploading your images, the radiologist is not going to look at them’”
	Rotating attendance schedule reduces MCC participant burnout	**✖**	✔	✔	“if we did have rotated priority that would be helpful, we are always kind of scrambling … we do have a sort of rotating schedule because if we don’t then someone is missing around somewhere”
	Adequate administrative supports improve efficiency of MCC	✔	✔	✔	“for years, we had every single brain tumor patient in the book written down, it was all in there and what the decision was, so it was a central repository … that is where you need administrator’s support”
Beliefs about Consequences	MCC allow for opportunities of learning	✔	☐	✔	“learning opportunities for residents could be improved”
	MCC empower physicians to make treatment decisions	✔	✔	n/a	“I had a contentious case where the patient is going to say, I want to see the multidisciplinary team’s opinion … in two cases I was able to show them (the MCC’s decision) and that stopped all of the argument”
	MCC can standardize decision-making for complex cases	✔	✔	n/a	“MCCs help create a framework for decision making”
	MCC allow physicians to obtain second opinions from colleagues efficiently	✔	✔	n/a	“it is just like having a curbside counsel with one of your colleagues”
	Decisions made in an MCC setting mitigate medico-legal risk	☐	☐	n/a	“You want insurance more than anything”
Reinforcements	CME (continued medical education) credits promote MCC participation	☐	☐	☐	“if you are relaxing on Friday and you have absolutely nothing to do then I think yeah then you would show up to get CME credits. And listen to the discussion. But I agree with you that I don’t think anybody because of that I think people who go because they get value out of getting their cases discussed” “getting CME credits for something that you do is always nice … but I don’t think anybody would go just because of that”
	Billing codes (i.e.: remuneration) promote MCC participation	☐	☐	☐	“we don’t get paid for it, but we don’t miss it, even if we have no cases to present … I billed for a case which is like $11” (implying that the billing is not an incentive) “lack of payments is a barrier” “everyone can bill except for pathologists. We treat it as our job and we are delighted to do it because there is no financial and we cannot bill because we are salaried”

While the validity of all themes were confirmed by some focus group participants, there were themes that did not generate consensus within and between focus groups. Table 5 demonstrates the differences in reported barriers and facilitators by focus group. For example, key informants identified the presence of bullying as a barrier. While some surgeon participants presented first-hand examples of when they were bullied, or treated poorly at rounds, others rejected the notion of bullying within their MCC team. Pathologists and radiologists did not report being personally bullied, but witnessed other treating physicians falling victim to negative group dynamics. In contrast, oncologists did not confirm this theme and did not perceive bullying or negative team dynamics to be a significant problem to MCC decision making.

#### Acceptability and appropriateness of KT-MCC strategy

The final study objective was to present the KT-MCC intervention components (workshops, team training, chair training, standard intake forms and synoptic checklist, and audit and feedback) to focus group participants to determine perceived acceptability and appropriateness. All groups identified chair training as an acceptable and appropriate intervention, as demonstrated in the following participant quote:

“*I think having an effective chair that helps promote flow and efficiency is very helpful. These things [leading a discussion] are not necessarily things that are intuitive.”*

Participants were also generally positive towards audit and feedback, use of a standard intake form, and the use of workshops to develop local consensus processes (i.e., ‘rules of functioning’ for MCCs). However, while they confirmed the appropriateness of team training and use of a synoptic discussion checklist (i.e., if implemented, would likely have a positive impact on MCC decision making) they were divided on whether the interventions were acceptable (i.e., ‘palatable’ or desirable). Some participants highlighted concerns that a synoptic discussion checklist would reduce the efficiency of a case discussion, and that limited time resources would preclude team training attendance, as demonstrated in the following quote,

*“Nobody is going to go (to a team training session). You can set it up and I don’t know if anybody would show up.”*

These perceptions were not consistent among team members and did not correlate with physician specialty group. For instance, other participants suggested that a team training session would improve soft-skills and team communication and would thus likely improve MCC efficiency.

## Discussion

TDF-rooted interviews identified numerous barriers and facilitators to optimal MCC decision-making. Identified themes were linked to TDF domains and then mapped to the COM-B to develop the first iteration of the KT-MCC Strategy which included workshops, team training, chair training, use of a standard intake form and synoptic discussion checklist, and, audit and feedback. Focus groups confirmed the validity of the key informant data and acceptability of the proposed KT-MCC Strategy interventions.

There are few examples in the KT literature that provide a complete and detailed description of how to design a complex KT strategy using the TDF and COM-B [48]. Even fewer studies evaluate the impact of such interventions on process or patient outcomes. As a result, there are limited examples in the implementation science literature to guide operationalization of the TDF and COM-B to develop pragmatic interventions. Existing examples include a comprehensive analysis by Alexander et al. using the TDF and COM-B to identify barriers to delivery of a health assessment for preschool children [49]. More recently, Garbutt et al. used the Consolidated Framework for Implementation Research (CFIR) followed by the TDF and COM-B to identify barriers to HPV vaccine guideline implementation and designed a multipronged intervention to overcome identified barriers [20]. We aim to add to this growing body of literature by presenting in detail our methods of mapping TDF domains to the Behaviour Change Wheel to develop the KT-MCC Strategy.

The purpose of the TDF is to guide researchers to identify domains that are most likely to impact a behavior change [48]. Our key informants revealed themes related to all domains, and we therefore incurred the challenge of interpreting themes pertaining to 14 TDF domains. A review of the KT literature did not identify any strategies to identify domains that were considered most salient by participants. Moreover, we were unable to determine whether some domains were more likely to influence MCC decision making, as compared to others. Our experience likely reflects those of other researchers who use the TDF to identify barriers to complex and multifaceted behaviours. Recently, Skolarus et al. proposed the use of discrete choice experiments (DCE) to allow for the prioritization of barriers and facilitators identified using the TDF [50]. Advancement in methods such as these will facilitate the identification of the most salient domains likely to influence a behavior of interest, as defined by the target population.

To confirm the validity of our TDF-guided key informant interviews, we held focus groups with MCC participants. Our focus group participants did not identify any new mediators to MCC decision making and did not recommend any additional interventions to those proposed. This reinforces the comprehensiveness of the TDF and COM-B to develop KT interventions. We encountered our second challenge in moving from COM-B intervention functions to pragmatic KT-MCC Strategy interventions. Once again, all facets of behaviour (COM) qualified for intervention, yet we were unable to determine whether some facets would hold a greater impact on behaviour than others. We opted for a multi-pronged approach that encompassed principles of all of the Behaviour Change Wheel intervention functions. To develop the details of each KT-MCC Strategy intervention component (e.g., frequency and content of team training session), we consulted Effective Practice and Organization of Care (EPOC) systematic reviews and other pertinent literature from multidisciplinary fields. Michie et al. are aiming to streamline such processes by developing an interactive online tool to link COM-B intervention functions to mechanisms of action known as Behaviour Change Techniques (BCT). BCTs represent the individual components (i.e., details) of intervention functions [31]. Explicit linkages between COM-B intervention functions, corresponding BCTs and evidence will significantly aid implementation scientists in the practical development of theoretically-rooted interventions.

Given that the acceptability of certain KT-MCC Strategy interventions differed by MCC participants, the intervention must be adaptable to individual MCC teams. To allow for such adaptations, we will implement the KT-MCC Strategy using an iterative, integrated knowledge translation approach – members of the target population (i.e., individual MCC teams) will modify the intervention to their own context, as warranted [51]. We will use an iterative approach to further tailor and refine the intervention. We posit that this approach will likely improve adoption of, and fidelity to, selected interventions.

To the best of our knowledge, this is the first study to utilize theory to identify domains that influence physician behaviour during MCC decision making processes. In 1995, the Calman-Hine report recommended a series of reforms to reduce inequality and improve outcomes for cancer care in the United Kingdom [52]. One of the key recommendations of the report was the promotion of multidisciplinary collaboration to improve the quality of cancer care. Since then, MCCs have been mandated for use or are commonly used in most North American and European countries [1, 2, 53]. Despite the increased emphasis and reporting on MCCs, there has been little evaluation of the quality of decision making at these rounds. We identified only one other example in the literature that implemented a quality improvement intervention that aimed to improve MCC decision making. The team, led by Lamb et al., introduced an intervention comprised of an MCC checklist, team training, and written guidance to optimize MCC decision making for a urological MCC in the United Kingdom [5]. The authors noted marginal improvements in the quality of teamwork and communication attributed to their intervention [5]. As the next step of our work, we will implement and evaluate the impact of the KT-MCC Strategy on the quality of MCC decision making.

Finally, qualitative literature regarding MCC quality mediators are generally consistent with our presented findings [2, 38, 53, 54]. For instance, Look Hong et al. completed a thematic analysis of key informant interviews with MCC physicians and administrators participating in Ontario gastrointestinal MCCs [2]. The study showed that efficient management of cases by a MCC chair, identification of strategies to improve participant attendance, and recognition of radiologist and pathologist roles were critical to MCC structure. Moreover, collegiality, level of teamworking, and the availability of technological and institutional factors were likely to impact MCC functioning. Similarly, Jalil et al. conducted a qualitative study in the United Kingdom to assess MCC participants’ views on decision making [54]. Their analysis indicated that inadequate clinical information, lack of investigation results, non-attendance of key members, and teleconferencing failures were major barriers to MCC decision making. While these findings are consistent with our results, our study provides the added advantage of exploring these themes comprehensively and as theoretical domains. This approach allowed us to directly map identified barriers and facilitators to corresponding KT interventions to develop the KT-MCC Strategy.

There are limitations to the presented study. First, key informant data may not be representative of MCCs across Ontario. Our research team aimed to promote generalizability of the findings by selecting a representative sample of physicians at multiple academic and community hospitals. As well, our findings are consistent with previously published MCC literature. Thus, our barriers and facilitators are likely generalizable to other jurisdictions. Second, the face validity of the KT-MCC Strategy was evaluated among focus group participants that represented a single LHIN. We posit that the findings presented in this study are likely generalizable beyond this LHIN, due to consistencies with our key informant data as well as Ontario and international MCC literature. However, in keeping with an integrated KT approach, individual Ontario MCC teams should adapt the KT-MCC Strategy prior to implementation. Third, this study demonstrated the comprehensiveness of using both the TDF and COM-B to design an implementation strategy; however, challenges with these methods were identified. Additional methods that can be used to prioritize barriers, facilitators and implementation strategies most likely to drive behaviour are needed.

## Conclusion

This is the first study to use a theoretical framework to identify barriers and facilitators to optimal MCC decision making, and to select corresponding interventions to develop a KT strategy designed to improve MCC decision making. Myriad processes influenced MCC decision making, as evidenced in our results. The developed KT-MCC Strategy included the use of workshops to identify team goals, team and chair training, standardized intake form and a synoptic discussion checklist, and audit and feedback. In the next phase of this study, we will pilot the KT-MCC Strategy to evaluate the feasibility of implementation and potential impact of the strategy on MCC decision making quality.

## Supplementary information

**Additional file 1.** Interview Guide.

## Data Availability

The data generated and analysed during the current study are available from the corresponding author on reasonable request.

## Abbreviations

BCT: Behaviour Change Techniques
CCO: Cancer Care Ontario
COM-B: Capabilities, Opportunities, and Behaviour – Behaviour Change Wheel
KT: Knowledge Translation
KT-MCC: Knowledge Translation-Multidisciplinary Cancer Conference Strategy (name of intervention)
LHIN: Local Health Integrated Network
MCC: Multidisciplinary Cancer Conference
TDF: Theoretical Domains Framework
