# Supplementary material for: Use of the theoretical domains framework and behaviour change wheel to develop a novel intervention to improve the quality of multidisciplinary cancer conference decision-making
Source: BMC Health Serv Res. 2020 Jun 24;20:578. doi: 10.1186/s12913-020-05255-w (PMC7313182; doi:10.1186/s12913-020-05255-w)
Supplement: Supplementary file 1 — Additional file 1. Interview Guide. [file 12913_2020_5255_MOESM1_ESM.docx]

Additional File 1: Interview Guide

| **TDF Domain** | **Questions** |
| --- | --- |
| **Knowledge** | 1. Our study is evaluating the impact of MCCs in Ontario. Are you aware of the CCO mandate regarding MCCs? *If not: state that every new or suspected cancer case should be discussed in a collaborative setting* 2. Do you know of any evidence to support this mandate? |
| **Skills** | 1. How easy or difficult do you find it to discuss every new or suspected case in a multidisciplinary cancer conference? |
| **Professional role and identity** | 1. Do you think that the CCO guideline should mandate behavior in real practice? 2. What are your perceptions of CCO guidelines, in general? 3. Do you believe that participating in a weekly multidisciplinary MCC limits physician autonomy? |
| **Beliefs about capabilities** | 1. Do you consider it easy or difficult to participate in MCCs? Why or why not 2. Have you encountered any problems by participating in MCCs? 3. Have you encountered any problems by not participating in MCCs? 4. What would help you attend MCCs more frequently OR if they attend, what would make the MCC process more enjoyable? 5. Are you comfortable discussing all of your treatment plans in a multidisciplinary context? |
| **Beliefs about consequences** | 1. Do you believe that discussing cases in a multidisciplinary context influences change in patient care? Why or why not? 2. Do you think there would be any consequences if you did not participate in MCCs OR Do you think that your colleagues who do not participate in MCCs face any consequences? 3. Do the benefits of MCCs outweigh the costs (costs to be prompted by conversation: ie lack of time, resources, etc.) |
| **Motivation and goals** | 1. Do you enjoy participating in MCCs? 2. Do you feel more comfortable with your management plans if they have been discussed in a multidisciplinary conference? 3. Which cases do you bring to MCC? How do you decide? 4. Are there any incentives to participating in MCC? Probe: are they aware that they can bill for MCCs? |
| **Memory, attention and decision processes** | 1. Are there any reasons why you do not/ would not participate in MCCs? (prompt: competing tasks, time constraints) 2. Do you believe that MCC generate consensus? Do you abide by this consensus/ by the group decision? Why or why not? Are there any factors that make you more likely to implement a decision re management (ie: who made the suggestion, evidence, etc.)? |
| **Environmental context and resources** | 1. To what extent do physical or resource factors provided by the hospital facilitate MCC participation? 2. To what extent do physical or resource factors provided by the hospital hinder MCC participation? |
| **Social influences** | 1. To what extent do social influences facilitate or hinder MCC participation? (peers, patients?) 2. Do the majority of your colleagues participate in MCCs? Does that influence your decision to participate? |
| **Emotion** | 1. Do MCCs lead to any tension between colleagues? Does this affect your decision to participate? 2. Do MCCs promote teamworking? Does this affect your decision to participate? |
| **Behavioural regulation** | 1. Is the manner by which MCCs are conducted helpful to case discussion? Why or why not? What could be improved? |
| **Nature of the behavior** | 1. How often should clinicians participate in MCCs (ie: should the same people show up every week, are there any consequences in that?) 2. Are there any systems in place/ what systems should be put in place to monitor the impact of MCCs? |
